# Novel Strategy for Phenotypic Characterization of Human B Lymphocytes from Precursors to Effector Cells by Flow Cytometry

**DOI:** 10.1371/journal.pone.0162209

**Published:** 2016-09-22

**Authors:** Giovanna Clavarino, Noémie Delouche, Claire Vettier, David Laurin, Martine Pernollet, Tatiana Raskovalova, Jean-Yves Cesbron, Chantal Dumestre-Pérard, Marie-Christine Jacob

**Affiliations:** 1 Laboratoire d’Immunologie, Département d’Hématologie, Oncogénétique et Immunologie, Pôle de Biologie, Grenoble University Hospital, Grenoble, France; 2 BNI, TIMC-IMAG, UMR 5525 CNRS, Grenoble, France; 3 Université Grenoble-Alpes, Grenoble, France; 4 Laboratoire d’Hématologie, Département d’Hématologie, Oncogénétique et Immunologie, Pôle de Biologie, Grenoble University Hospital, Grenoble, France; 5 TheREx, TIMC-IMAG, UMR 5525 CNRS, Grenoble, France; 6 Etablissement Français du Sang Rhônes-Alpes Auvergne, La Tronche, France; 7 CNRS UMR 5309 and INSERM U1209, Institut Albert Bonniot, Grenoble, France; Harvard Medical School, UNITED STATES

## Abstract

A precise identification and phenotypic characterization of human B-cell subsets is of crucial importance in both basic research and medicine. In the literature, flow cytometry studies for the phenotypic characterization of B-lymphocytes are mainly focused on the description of a particular cell stage, or of specific cell stages observed in a single type of sample. In the present work, we propose a backbone of 6 antibodies (CD38, CD27, CD10, CD19, CD5 and CD45) and an efficient gating strategy to identify, in a single analysis tube, a large number of B-cell subsets covering the whole B-cell differentiation from precursors to memory and plasma cells. Furthermore, by adding two antibodies in an 8-color combination, our approach allows the analysis of the modulation of any cell surface marker of interest along B-cell differentiation. We thus developed a panel of seven 8-colour antibody combinations to phenotypically characterize B-cell subpopulations in bone marrow, peripheral blood, lymph node and cord blood samples. Beyond qualitative information provided by biparametric representations, we also quantified antigen expression on each of the identified B-cell subsets and we proposed a series of informative curves showing the modulation of seventeen cell surface markers along B-cell differentiation. Our approach by flow cytometry provides an efficient tool to obtain quantitative data on B-cell surface markers expression with a relative easy-to-handle technique that can be applied in routine explorations.

## Introduction

A precise identification of human B-cell subpopulations is of pivotal importance in both basic research and medicine. In human, B-cell differentiation takes place in two main locations. After birth, B-cell lymphopoiesis is occurring in the bone marrow from B-cell precursors (or hematogones) to transitional B-cells that migrate out of the marrow into the peripheral blood. This first phase of B-cell development is antigen independent and leads to B-cells having a functional membrane B-cell receptor [1]. The second phase of B-cell differentiation, driven by antigen stimulation, takes place in peripheral lymphoid organs and leads to memory cells or plasma cells [2] [3]. This differentiation and maturation of B lymphocytes can be monitored by changes in cytomorphologic, genetic, molecular and immunophenotypic characteristics. Along B-cell differentiation, some surface or intracellular proteins are newly expressed or up regulated, whereas others are down regulated and even disappear [4].

Using multiparametric flow cytometry, variations of phenotypic markers can clearly be observed, and multiple stages of B-cell lymphopoiesis can be defined based on their immunophenotype [5] [6] [7]. However, phenotypic studies are often focused on a particular type of sample (bone marrow, peripheral blood, lymphoid organs, cord blood) [8] [9] [10] or on a particular B-cell subset [11] [12] [13] [14] [15]. Fine examples of B-cell differentiation analysis are the studies, in the early 2000’s, by van Lochem in bone marrow [8] or Bohnhorst in lymph nodes [9]; however, only four-colour combinations were used for the delineation of only few stages of maturation. Multicolour panels for phenotypic analysis of B and plasma cells have recently been proposed, but only in rhesus macaques [16]. Recently, a strategy combining single-cell mass cytometry with a computational algorithm, allowed the construction of a human B-lineage trajectory representing in vivo development from B-cell precursors in the bone marrow to naive B cells [17]. So far, a routinely usable strategy allowing the phenotypic characterization of B-cell subpopulations throughout B-cell differentiation in samples from different anatomical sites has not been reported in human, using flow cytometry.

A first objective of the present work was to identify a maximum number of B-cell subsets with a minimal number of antibodies. Indeed, with a backbone of six antibodies (CD38, CD27, CD10, CD19, CD5 and CD45), we could identify seven B-cell subsets from precursors to effector cells, i.e. stage 1 hematogones, precursors B cells including stage 2 hematogones and immature B cells, transitional B cells, naive B cells, germinal center B cells, memory B cells, and plasma cells. By adding in the same tube two antibodies against IgM and IgD, other subsets could be discriminated: immature B-cells (IgM^+^ IgD^-^) could be separated from stage 2 hematogones (IgM^-^ IgD^-^) within precursor B-cells, and natural memory B-cells (IgM^+^ IgD^+^) could be distinguished from post-germinal center switched (IgM^-^ IgD^-^) or unswitched (IgM^+^IgD^-^) memory B cells within mature B-cells. Therefore, a total of ten B-cell subsets could be identified in one single 8-color tube.

The possibility to replace IgM and IgD antibodies with two different antibodies makes our combination flexible, allowing the analysis of the modulation of any surface marker of interest along B-cell differentiation pathway. Thus, a second objective of our study has been to develop a panel of seven 8-color tubes in order to phenotypically characterize B-cell subpopulations in different anatomical sites such as bone marrow, peripheral blood, lymph node and cord blood samples. Phenotypic B-cell characterization was thus explored by biparametric analysis, as well as by antigenic quantification, providing informative curves representing the median fluorescence intensities of seventeen markers expressed on the main subsets of the B-cell lineage. Thus, the use of flow cytometry allows obtaining quantitative data with a relative easy-to-handle technique, thereby possible to employ in routine explorations.

## Materials and Methods

### Patients and biological samples

Three peripheral blood samples from 2, 25 and 27 year-old individuals (two males and one female, respectively) without any hematological pathology and three cytomorphologically normal bone marrow samples from 5, 38 and 58 year-old individuals (two females and one male, respectively) were collected in EDTA-containing tubes (Becton Dickinson). Mononuclear cells were separated by density gradient centrifugation using lymphocyte separation medium (Eurobio) 24 h maximum after sample collection. Antibody staining and cytometry analysis were conducted right after. Three lymph node samples with reactive hyperplasia from 18, 43 and 58 year-old patients (two females and one male, respectively) were also selected. Cells were kept at 4°C in RPMI (Life Technology) supplemented with 2% fetal calf serum, penicillin and streptomycin for a maximum of 18 hours, then gently dissociated with a scalpel, filtered through a 100μm cell strainer to remove aggregates, and washed twice [18]. Routine analysis was then performed, and remaining cells were kept at 4°C in Hank’s balanced salt solution with 2% of fetal calf serum in order to preserve cell viability. The maximum delay between extraction and analysis on the cytometer was 48 hours. All samples were obtained from patients from Grenoble University Hospital. For all these samples, we used the remaining material after routine immunophenotyping. The study was done in accordance with the Declaration of Helsinki and Good Clinical Practice guidelines, and it was approved by the Institutional Review Board (“Commission Nationale de l’Informatique et des Libertés”, CNIL N°1981710v0). Patients were informed and non-opposition was obtained according to French law. Furthermore, three mononuclear cell samples from cord blood (male individuals) frozen in liquid nitrogen were obtained from the French Blood Establishment in Grenoble (declared biobank TCG/15/R/001; patient’s parents provided written consent) and thawed just before staining and cytometry analysis; also in this case, we used the remaining material after routine investigations.

Cells from all samples were counted with an ABX Micros 60 (Horiba Medical) instrument.

### Immunofluorescence staining and flow cytometry analysis

Phenotypic analysis of cell suspensions was performed by flow cytometry. Cells were stained with a panel of seven 8-colour antibody combinations (Table 1). The following 6 monoclonal antibodies were used as a “backbone” in all combinations: CD38-PerCP5.5, CD10-APC, CD19-APC-H7, CD5-V450, CD45-V500 (BD Biosciences) and CD27-PeCy7 (Beckman Coulter). All FITC- or PE-labelled antibodies were specific of a particular combination, i.e. CD20-FITC, CD44-FITC, CD24-PE, CD40-PE, (Beckman Coulter); CD43-FITC, CD81-FITC, CD86-FITC, CD21-PE, CD22-PE, CD23-PE, CD268 (BAFF-R)-PE, IgD-PE, (BD Biosciences) IgM-FITC (Dako). Clone and isotype specificity of these antibodies are detailed in S1 Table; the antibodies were used at the dilution recommended by the manufacturers.

**Table 1:** Panel of seven 8-colour antibody combinations.

Fluorochromee	FITC	PE	PerCP-Cy5.5	PE-Cy7	APC	APC-H7	V450	V500
Tube								
1	IgM	IgD	**CD38**	**CD27**	**CD10**	**CD19**	**CD5**	**CD45**
2	CD44	CD24	**CD38**	**CD27**	**CD10**	**CD19**	**CD5**	**CD45**
3	CD81	CD22	**CD38**	**CD27**	**CD10**	**CD19**	**CD5**	**CD45**
4	CD20	CD23	**CD38**	**CD27**	**CD10**	**CD19**	**CD5**	**CD45**
5	CD43	CD21	**CD38**	**CD27**	**CD10**	**CD19**	**CD5**	**CD45**
6	CD86	BAFF-R	**CD38**	**CD27**	**CD10**	**CD19**	**CD5**	**CD45**
7	X	CD40	**CD38**	**CD27**	**CD10**	**CD19**	**CD5**	**CD45**

Antibodies forming the common backbone for all the tubes are on a grey background; FITC- or PE-labelled antibodies are specific of a particular combination.

Samples containing 10^6^ cells in a volume of 100 μl were incubated with the combination of antibodies at the supplier recommended concentration during 20 minutes in the dark, at 4°C. After erythrocytes lysis with NH_4_Cl at 4°C for 5 minutes, samples were washed with HBSS medium (Gibco).

Analysis was performed using 3-laser, 8-colour BD FACSCanto II flow cytometer (BD Biosciences) and FACSDiva software version 6 (BD Biosciences). At least 10^4^ cells were acquired in the CD19^+^ gate. BD CompBeads (BD Biosciences) were used for compensation settings. Cytometer performances were checked daily using CST beads (BD Biosciences).

## Results

### Identification of up to ten B-cell subsets using a unique combination of eight antibodies

**Fig 1:**
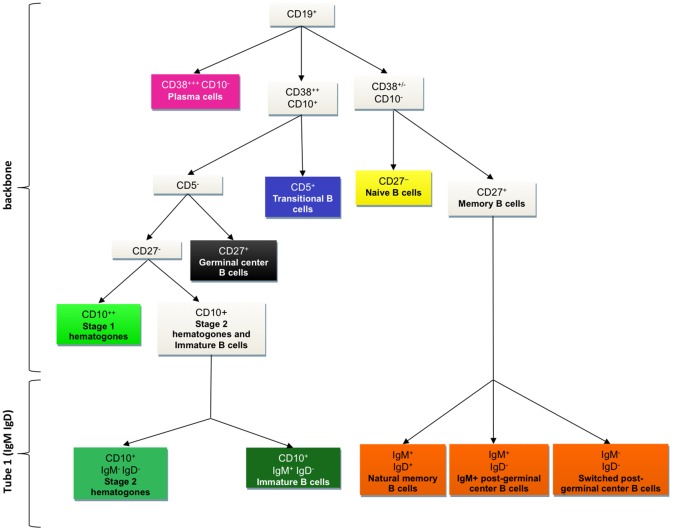
Strategy of identification of the B-cell subpopulations with the backbone antibodies (CD38, CD27, CD10, CD19, CD45, CD5) and IgM and IgD markers.

**Fig 2:**
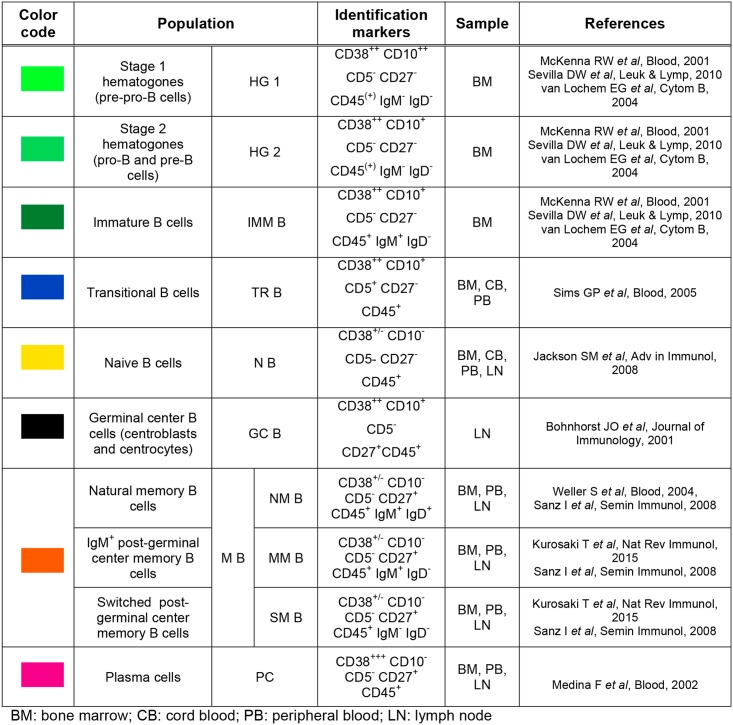
Phenotypic chatacterization of B-cell subsets.

The use of the backbone of the following six antibodies CD38, CD27, CD10, CD19, CD5 and CD45 (Table 1), allowed us to establish a strategy for the phenotypic identification of seven major B-cell subsets (Figs 1 and 2). CD19 was chosen to select B cells, because of its positivity all along the lineage from the most immature precursors up to memory and plasma cells; the other antibodies optimized the discrimination of such subsets.

The differential expression of CD38 and CD10 surface markers on CD19^+^ B cells distinguished three populations: the first CD38^+++^ CD10^-^ subset consists uniquely of plasma cells that express the highest level of CD38. To note, all plasma cells are thus selected, without discrimination of their different stages of maturation. In contrast, the second population of CD38^++^ CD10^+^ cells is heterogenous: cells express the same level of CD38, but they can be first discriminated according to CD5 expression. Among these cells, those expressing CD5 are transitional B cells [14], while those lacking CD5 are still heterogeneous, and can be further subdivided, according to the expression of CD27, into CD27^-^ hematogones and CD27^+^ germinal center B cells. Furthermore, depending on the intensity of CD10, hematogones can be separated into strongly positive stage 1 and moderetely positive stage 2 hematogones and immature B cells [19] [20]. It is to note that stage 1 hematogones correspond to pre-pro-B cells precursors and stage 2 hematogones correspond to pro-B and pre-B cells precursors [17]. Finally, the third group of CD38^+/-^ CD10^-^ cells includes naive B cells (CD27^-^) and memory B cells (CD27^+^).

**Fig 3:**
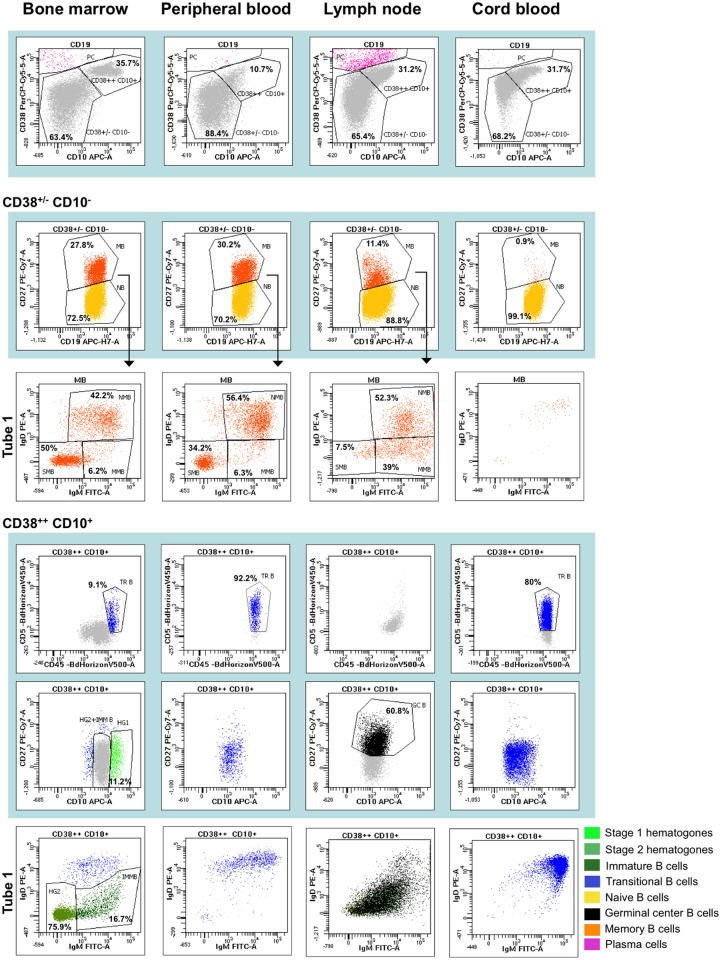
Gating strategy for B-cell subsets’ identification. Dot plots on blue background identify subpopulations from the antibody backbone. HG 1: hematogones stage 1, HG 2: hematogones stage 2, IMM B: immature B cells, TR B: transitional B cells, N B: naive B cells, GC B: germinal center B cells, M B: memory B cells, NM B: natural B memory cells, MM B: non-switched memory B cells, SM B: switched memory B cells, PC: plasma cells.

Using the two additional antibodies against surface IgM and IgD in the first tube of the panel (Table 1), immature B cells (IgM^+^ IgD^-^) could be distinguished from stage 2 hematogones (negative for both IgM and IgD) within CD38^++^ CD10^+^ CD5^-^ B-cell precursors. Moreover, memory B cell subset could be further divided in natural B memory cells IgM^+^ IgD^+^ [21] and post-germinal center B cells, including unswitched lymphocytes (IgM^+^ IgD^-^) and switched lymphocytes (IgM^-^ IgD^-^) (Fig 1) [22]. This leads to a total of ten B-cell subsets in one tube. The gating strategy allowing the identification of B-cell subsets from the antibody backbone, IgM and IgD is detailed in Fig 3.

### Quantification of B-cell subsets in bone marrow, peripheral blood, lymph node and cord blood samples

**Fig 4:**
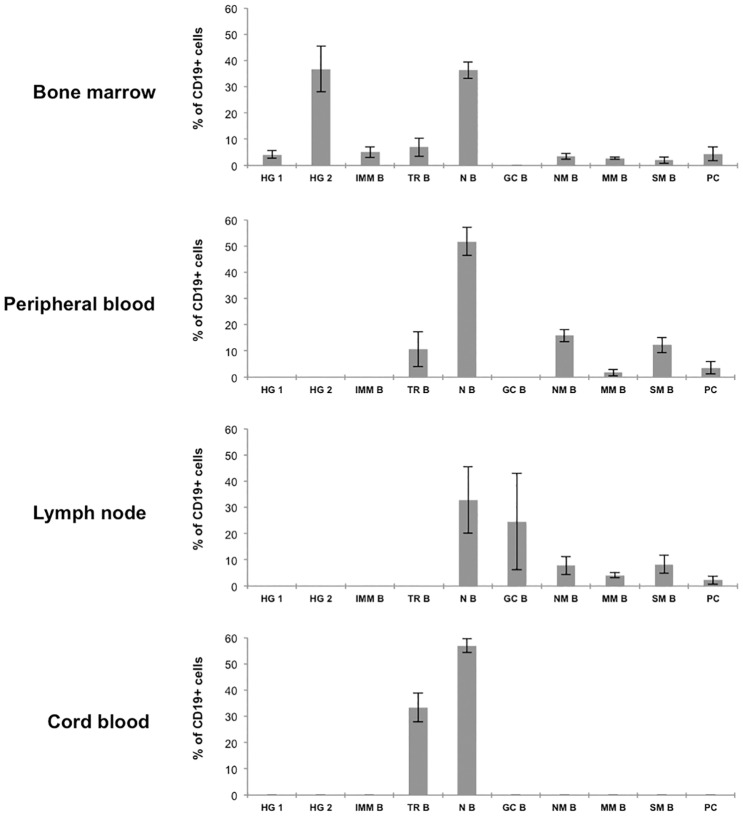
Graphical representation of the frequency (mean ± SD) of each stage of B-cell differentiation identified in the different types of samples. Samples of different anatomical sites (bone marrow, peripheral blood, lymph node and cord blood) were stained with the antibody combination of tube 1 of the panel and analyzed with the gating strategy shown in Fig 2 in order to identify ten B-cell subsets. Cell frequency has been quantified by calculating the mean of % of cells of each subset within CD19^+^ cells (± standard deviation). HG 1: hematogones stage 1, HG 2: hematogones stage 2, IMM B: immature B cells, TR B: transitional B cells, N B: naive B cells, GC B: germinal center B cells, NM B: natural B memory cells, MM B: non-switched memory B cells, SM B: switched memory B cells, PC: plasma cells. Data from three patients for each type of sample are represented.

In order to evaluate the validity of the method, we applied this gating strategy to quantify the frequency of B-cell subsets in samples of various origins -bone marrow (n = 3), cord blood (n = 3), peripheral blood (n = 3) and lymph node (n = 3)- (Fig 4). Age and characteristics of patients have been detailed in materials and methods section. As expected, in bone marrow all B-cell subpopulations, with the exception of germinal center B cells, were identified: stage 1 and stage 2 hematogones, immature B cells, transitional B cells, naive B cells, natural and post-germinal center memory B cells (including switched and unswitched B memory cells) and plasma cells [8]. Naive and memory B cells were likely from peripheral blood due to sample dilution. In cord blood samples, we essentially found transitional and naive B cells [10]. In peripheral blood, we identified all B-cell subsets from transitional stage to the plasma cells, with the exception of germinal center B cells; no B-cell precursors were observed [4, 12, 14]. Among B memory lymphocytes, natural memory B cells and post-germinal center memory B cells, switched and unswitched, could be discriminated. In the lymph nodes, B-cell subpopulations were observed starting from naive B-cells up to memory B-cells and plasma cells [9, 12].

As a demonstration of the feasibility of our approach, we also showed some examples of applications. We illustrated how B-cell subsets vary with the age of the subjects. As expected, transitional B-cells are decreasing, whereas post-germinal center memory B cells are increasing in blood samples from children to adults (S1A Fig). Likewise, we showed the diminution of hematogones in bone marrow from children to adults (S1B Fig). We also showed two cases of immune reconstitution after stem cell transplantation. Interestingly, the analysis of B-cell subsets revealed that mature B cells are not present in either peripheral blood or bone marrow samples at early time after transplantation (S2 Fig).

### Phenotypic characterization of B cells during maturation

**Fig 5:**
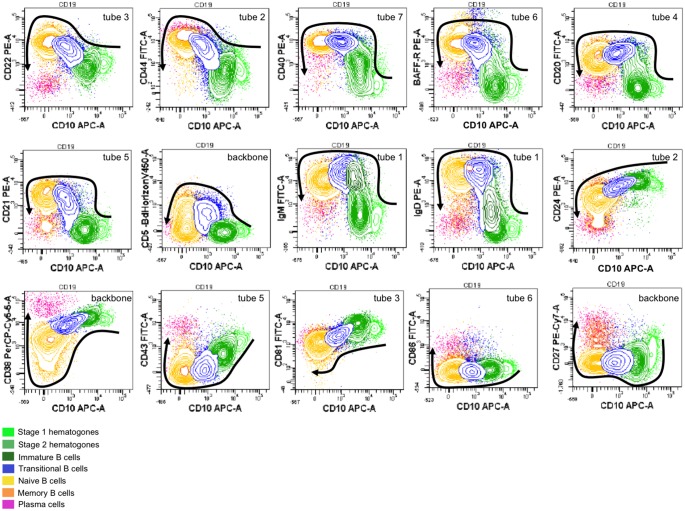
Biparametric representations of B-cell maturation pathways in the bone marrow. The modulation of the expression of various cell surface markers versus CD10 expression is represented in dot plots. The specific tube of antibody combination used to analyze the expression of each marker is indicated in each graph, at the upper right. B-cell maturation is shown with arrows, drawn from the most immature B-cell subset to the most mature one. Data from one patient out of three with similar results are here represented.

**Fig 6:**
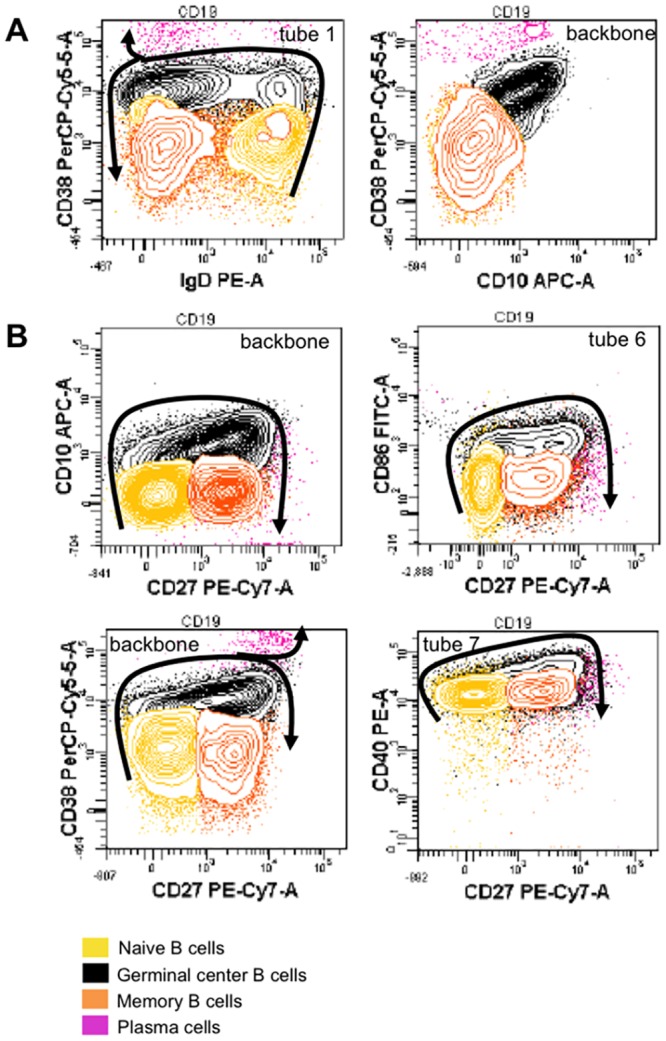
Biparametric representations of B-cell maturation pathways in the lymph node. Identification of B-cell subsets in lymph node (A) and analysis of the expression of various cell surface markers versus CD27 expression (B). The specific tube of antibody combination used to analyze the expression of each marker is indicated in each graph, at the upper right. B-cell maturation is shown with arrows, drawn from the most immature. Data from one patient out of three with similar results are here represented.

**Fig 7:**
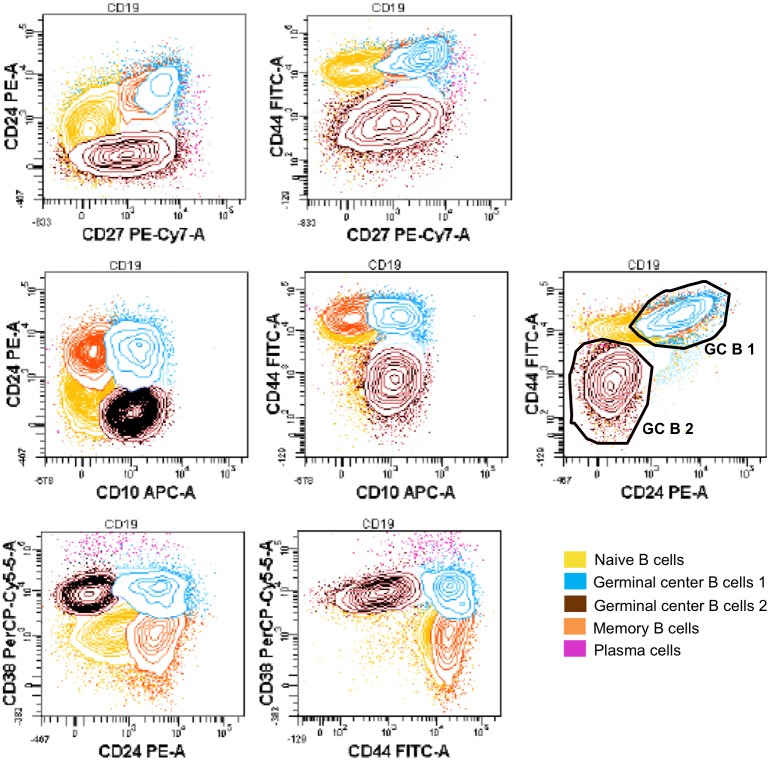
CD24 and CD44 expression during B cell differentiation in lymph nodes. CD24 and CD44 markers define two separate subsets within germinal center B cells. Data from one patient out of three with similar results are here represented.

As previously mentioned, a combination of six antibodies allowed the identification of seven B-cell subsets from precursors up to effector cells. Using a panel of seven tubes with two additional antibodies to this backbone (Table 1), we could analyze the modulation of expression of surface antigens on these different B-cell subsets in bone marrow (Fig 5) and in lymph node (Figs 6 and 7).

In bone marrow, we chose CD10 as a constant parameter to characterize B-cell maturation, since its expression decreased gradually from hematogones to transitional B cells, and disappeared at the stage of naive B cells; the expression of all other markers was thus presented versus CD10 expression (Fig 5). We thus analyzed the expression of several cell surface antigens: CD22 and CD44 were already present at the earliest stage of differentiation, whereas CD40 and BAFF-R appeared later, followed by CD20 and then CD21. All of them were upregulated during differentiation and have disappeared at the stage of plasma cells. CD5 also appeared late, but its expression was transient, characterizing the stage of transitional B cells. We also clearly showed that the appearance of IgD at the cell surface is delayed compared to IgM. On the contrary, the expression of CD24, CD38, CD43 and CD81 was very high on precursor’s surface and progressively decreased during maturation; only CD38 and CD43 were upregulated at plasma cell stage.

In lymph node, the main B-cell subsets could be identified using CD38 versus IgD analysis, as in Bohnorst’s study [9] (Fig 6A). Naive B cells were CD38^low^ IgD^+^; then, CD38 progressively increased, while IgD was lost when B cells entered the germinal center. On the way of differentiation into memory cells, IgD remained negative and CD38 progressively decreased, whereas on the way of differentiation into plasma cells, CD38 was even more upregulated. We could show that CD10 expressing cells actually matched CD38^high^ subsets, thus confirming to be germinal center B cells (Fig 6A). Interestingly, we also showed the upregulation of CD27 expression during the differentiation of B-cells in the germinal center. Since CD27 was still expressed on memory and plasma cells, it was a very useful marker in order to study B-cell differentiation and analyze the sequential acquisition of different antigens in lymph node. Thus, we could observe that CD10, CD86 and CD38 appeared before the expression of CD27, whereas CD40 and CD27 increased simultaneously (Fig 6B). CD24 and CD44 were both expressed on naive B cells. Interestingly, we clearly observed that they defined two separate subsets within germinal center B cells (Fig 7): one subset is positive for both markers, while the other subset is negative for both of them. Memory B-cells revealed to be CD44^+^ CD24^+^, whereas plasma cells were CD44^+^ CD24^-^; since CD24 was not expressed on plasma cells, this surface marker could possibly predict the orientation of germinal center B cells towards differentiation into B memory cells or plasma cells.

### Quantitative evaluation of cell surface marker expression during B-cell maturation

**Fig 8:**
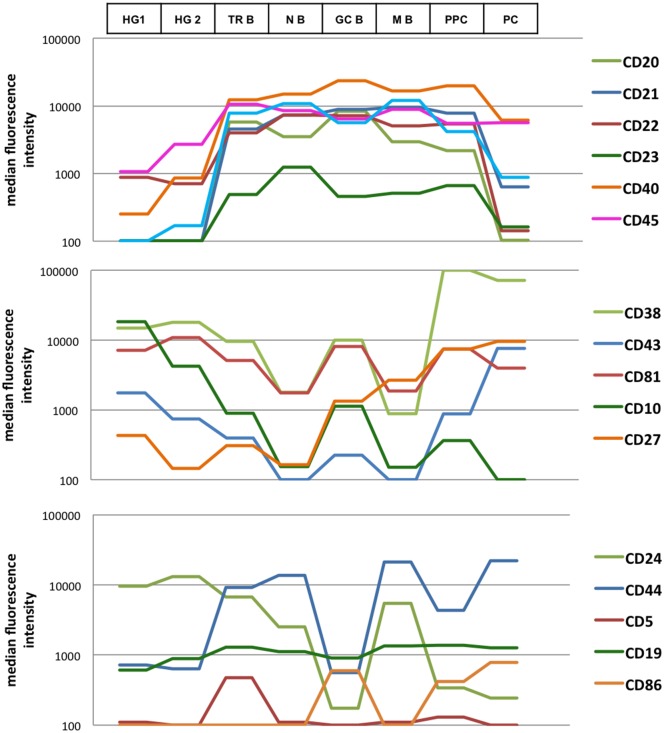
Relative expression patterns of 17 B-cell markers based on median fluorescence intensities (MFI). Means of MFI of three stage 1 or 2 hematogone samples, nine transitional B cell samples, twelve naive B cell samples, three germinal center B cell samples, nine memory B cell samples, six plasma cell samples are represented. Expression levels between different markers cannot be compared, since the measured intensity of fluorescence depends also on the specific fluorochrome bound to the antibody. HG 1: hematogones stage 1, HG 2: hematogones stage 2, TR B: transitional B cells, N B: naive B cells, GC B: germinal center B cells, M B: memory B cells, PPC: pre-plasma cells, PC: plasma cells.

The expression of a total of seventeen markers was quantified using median fluorescence intensity values (MFI) on all B-cell subsets in each sample (Fig 8). To note, each value represents the mean of all MFI for a given subset from all samples: thus, the number of measurements varies from three (in the case of hematogones, immature B cells and germinal center B cells) to six (for plasma cells), nine (for transitional and memory B cells) or twelve (for naive B cells). We thus provided informative curves showing the modulation of each antigen along B-cell differentiation from the hematogone stage to plasma cells (Fig 8). It should be pointed out that if it was possible to compare the expression of a given marker throughout B cell differentiation, this was impossible between different markers, since the MFI values depend also on the characteristics of the specific fluorochrome bound to the antibody. The calculation of standard deviations of MFI values for each marker demonstrated a homogeneous expression at each stage of differentiation, whatever the localization of the subset, with the exception of plasma cells (S3 Fig). In fact plasma cells in lymph nodes and blood samples are precursors (pre-plasma cells) of fully differentiated bone marrow plasma cells, with substantial phenotypic differences [23]. Thus, these two subsets have been separated in our analysis. Pre-plasma cells were CD20^low^, CD21^+^, CD22^+^, CD40^+^, CD86^low^, whereas plasma cells were CD20^-^, CD21^-^, CD22^-^ and CD86^+^ (Fig 8 and S3 Fig).

Our results revealed that, among the seventeen markers quantified, CD20, CD21, CD22, CD23, CD40, CD45 and BAFF-R showed a similar modulation: their expression increased gradually from the hematogone stage throughout B-cell maturation and then decreased at the plasma cell stage. CD38, CD43, CD81 and CD10 also showed similar modulation, but inversely to the previously mentioned antigens: their expression first decreased from hematogones to naive B cells, then transiently increased in the germinal center, and finally allowed to distinguish memory cells (low or no expression) from plasma cells (high expression); only CD10 was negative on both subsets. Other markers showed more complex variations. CD27 was negative until its expression on germinal center B cells, where it increased importantly up to memory B cell and plasma cell stages. CD24 progressively decreased from hematogones to the germinal center stage; it was expressed on memory B cells and not on plasma cells. CD44 was expressed all along the differentiation, with the lowest level on B-cell precursors and germinal center; CD5 was particularly expressed on transitional B cells; CD86 was found mainly on germinal center B cells and plasma cells; CD19 expression increased very gradually at B-cell precursor stages and then it remained stable.

## Discussion

In the present work, we developed a 8-color panel of antibodies as well as a robust analysis strategy with the aim of precisely studying B-cell lineage. Data from the literature are sufficiently well validated to base the identification of B-cell subsets on phenotypic characteristics (Fig 2).

The first combination of antibodies (IgM / IgD / CD38 / CD27 / CD10 / CD19 / CD5 / CD45), was devoted to discriminate a high number of B-cell subsets all along their maturation pathway from precursors to effector cells, in order to characterize the cell composition of any biological sample. Using a complex, but readily reproducible, hierarchical gating strategy, we could identify ten B-cell subsets, which well described early maturation in bone marrow, and antigen-driven differentiation in secondary lymphoid organs. This contrasts with other reports from the literature, which developed panels that are focused on phenotypic characterization of a particular cell stage [19], or of different cell stages in a single type of sample such as bone marrow [8] [17], peripheral blood [21] or lymph nodes [9]. In our work, the cell composition of various anatomical sites could adequately be described, with respectively nine, two, six and seven identified B-cell subsets in bone marrow, cord blood or peripheral blood and lymph nodes. This strategy proved to be correct, since the expected populations were actually recognized in the proper anatomical sites. For example, B-cell precursors were exclusively found in bone marrow, and germinal center B cells in lymph nodes only. Likewise, only naive and transitional B-cells were revealed in cord blood samples, whereas all sets of memory B-cells were present in adult peripheral blood. The use of 8-color panel allows application in any medical laboratory equipped with current flow cytometers, using the most commonly employed software such as DIVA.

A precise identification of human B-cell subsets in biological samples is of crucial importance in pathological conditions. It should be pointed out that normal B-cell count do not imply necessarily an adequate immunity; thereby, it has been proved to be important to quantify subsets such as transitional B cells, which can be abnormally predominant in peripheral blood explaining the failure of immune functions, as it is well documented in secondary immunodeficiencies due to HIV [24], hematopoietic stem cell transplantation [25] or after rituximab treatment [26] [27]. Also, our strategy could precisely identify the blockage of differentiation in primary immunodeficiencies, such as Bruton agammaglobulinemia, X-linked lymphoproliferative disease [28], hyper-IgM syndrome or provide reliable prognostic parameters in the case of common variable immunodeficiency, by quantifying transitional and post germinal center B-cells [29]. Studying the variation of particular B-cell subsets could also be interesting in the follow up of patients receiving immunomodulating molecules such as vaccines or interferons [30].

The possibility to add two antibodies to the common backbone, makes our panel flexible, in order to follow the modulation of any antigen of interest throughout B-cell differentiation. B-cell differentiation in bone marrow and lymph nodes has already been described in the literature [8] [31] [9]; however, only four-color combinations were used for the delineation of only few stages of maturation. In the present work, the sequential upregulation of CD22, CD20, IgM and IgD is nicely observable during early maturation in bone marrow samples; similarly, the expected downregulation of CD24, CD38 and CD10 markers is also clearly visible [11] [8]. In lymph node, germinal center B cells are identified as CD38^high^ CD10^+^ CD27^+^ cells [32] [9], and maturation from naive B cells (CD38^low^ CD10^-^ CD27^-^) towards memory B cells (CD38^low^ CD10^-^ CD27^+^) or plasma cells (CD38^high^ CD10^-^ CD27^+^) could distinctly be followed [33]. To note, our approach revealed interesting insights concerning CD24 marker. While it is well documented that CD24 and CD44 are downregulated in germinal center [9] [33], our data clearly demonstrate the existence of two distinct subsets among germinal center B cells, i.e. CD24^+^ CD44^+^ and CD24^-^ CD44^-^ cells. Since memory B cells are CD24^+^ and plasma cells CD24^-^, we hypothesize that the orientation towards these two subsets takes place early in the germinal center, and that CD24 might be a marker allowing the prediction of the further differentiation pathway of germinal center B cells.

Besides qualitative information provided by biparametric representations, we also quantified antigen expression on each of the identified B-cell subsets by measuring the median value of fluorescence, and we propose a series of informative curves showing the modulation of seventeen antigens along B-cell differentiation. Recently, the median level of an important number of B-cell markers was simultaneously measured by single-cell mass cytometry in bone marrow, and the rise and fall of phenotypic markers along a developmental trajectory obtained by a computational algorithm has been represented [17]. Interestingly, our work confirms the results obtained in this study; in addition, our investigation is extended up to B memory cell subsets and plasma cells.

## Conclusions

The unique combination of antibodies and the gating strategy that we developed may be of great interest in both basic research and medicine, a lot of information being provided even for very small cell samples. Furthermore, the use of only 8-color multiparametric analysis, allows application in any laboratory equipped with at least 8-color flow cytometers.

## Supporting Information

S1 Fig B-cell subsets vary with the age of the subjects. Transitional B-cells are decreasing whereas post-germinal center memory B cells are increasing in blood samples from children to adults (subjects were a two-month-old male, a two-year-old male, a twelve-year-old female and a forty five-year-old male). Frequencies of subsets showed in the table have been calculated within CD19^+^ cells (S1A Fig). Likewise, hematogones decrease in bone marrow from children to adults (subjects were a one-year-old male and a twenty-year-old female) (S1B Fig).
(PDF)

S2 Fig Immune reconstitution of transitional B cells in peripheral blood (dot plots shown are from two different three-year-old male subjects) and in bone marrow (dot plots shown are from two different twenty-year-old female subjects) after stem cell transplantation. Mature B cells are not present in either peripheral blood or bone marrow samples at early time after transplantation.
(PDF)

S3 Fig Expression of 17 cell surface markers in B-cell subsets in bone marrow (n = 3), peripheral blood (n = 3), lymph node (n = 3) and cord blood (n = 3) samples (means of medians of fluorescence intensities ± standard deviations). (PDF)

S1 Table Characteristics of antibodies. (PDF)
