# Supplementary material for: Novel Strategy for Phenotypic Characterization of Human B Lymphocytes from Precursors to Effector Cells by Flow Cytometry
Source: PLoS One. 2016 Sep 22;11(9):e0162209. doi: 10.1371/journal.pone.0162209 (PMC5033467; doi:10.1371/journal.pone.0162209)

A

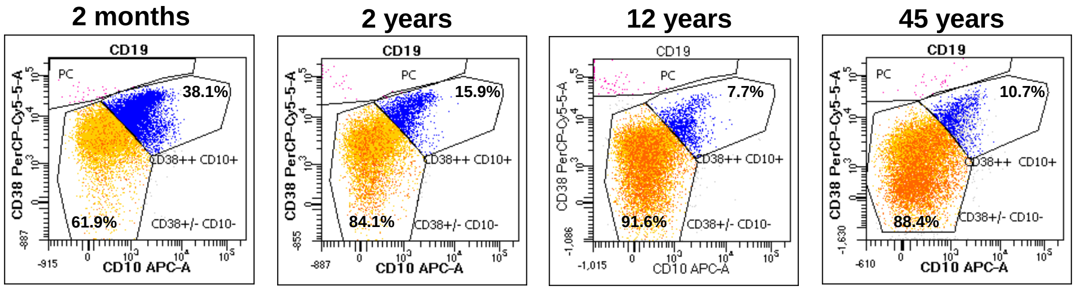

Frequency within B cells	Naive B cells	Memory B cells	Natural memory B cell	IgM$^+$ post-germinal center memory B cells	Switched post-germinal center memory B cells
**2 months**	59%	3%	3%	0%	0%
**2 years**	75%	9%	3,9%	1,7%	3,4%
**12 years**	73%	19%	7,5%	2,5%	9%
**45 years**	62%	27%	15%	1,7%	10,3%

B

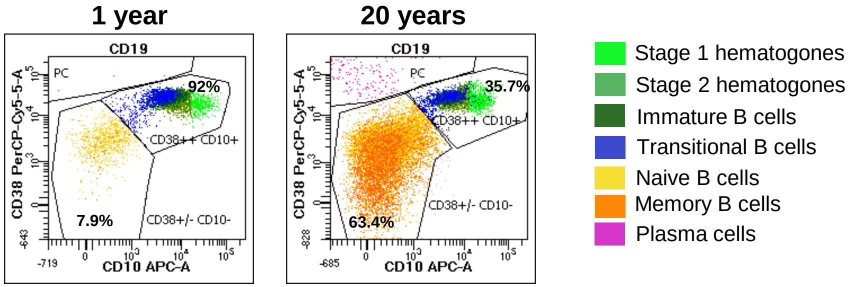

Supplement: S1 Fig — Transitional B-cells are decreasing whereas post-germinal center memory B cells are increasing in blood samples from children to adults (subjects were a two-month-old male, a two-year-old male, a twelve-year-old female and a forty five-year-old male). Frequencies of subsets showed in the table have been calculated within CD19+ cells (S1A Fig). Likewise, hematogones decrease in bone marrow from children to adults (subjects were a one-year-old male and a twenty-year-old female) (S1B Fig). (PDF) [file pone.0162209.s001.pdf]
