# Supplementary material for: Novel Strategy for Phenotypic Characterization of Human B Lymphocytes from Precursors to Effector Cells by Flow Cytometry
Source: PLoS One. 2016 Sep 22;11(9):e0162209. doi: 10.1371/journal.pone.0162209 (PMC5033467; doi:10.1371/journal.pone.0162209)
Supplement: S2 Fig — Mature B cells are not present in either peripheral blood or bone marrow samples at early time after transplantation. (PDF) [file pone.0162209.s002.pdf]

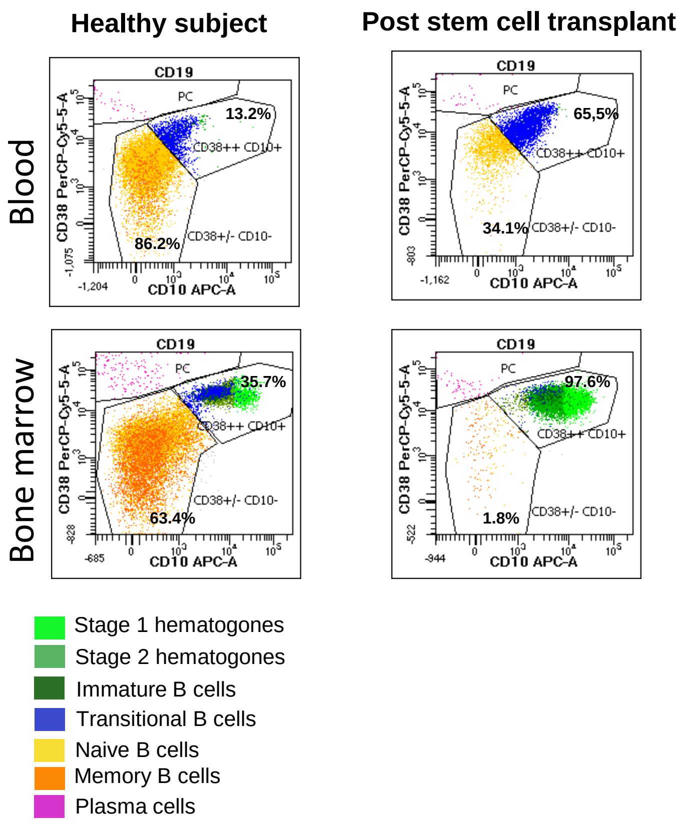
Healthy subject
Post stem cell transplant
Blood
Bone marrow
CD19
PC
13.2%
CD38++ CD10+
86.2%
CD38+/- CD10-
65,5%
34.1%
35.7%
63.4%
97.6%
1.8%
CD38 PerCP-Cy5-5-A
CD10 APC-A
Stage 1 hematogones
Stage 2 hematogones
Immature B cells
Transitional B cells
Naive B cells
Memory B cells
Plasma cells
