# Supplementary figures and images for: Novel Strategy for Phenotypic Characterization of Human B Lymphocytes from Precursors to Effector Cells by Flow Cytometry
Source: PLoS One. 2016 Sep 22;11(9):e0162209. doi: 10.1371/journal.pone.0162209 (PMC5033467; doi:10.1371/journal.pone.0162209)

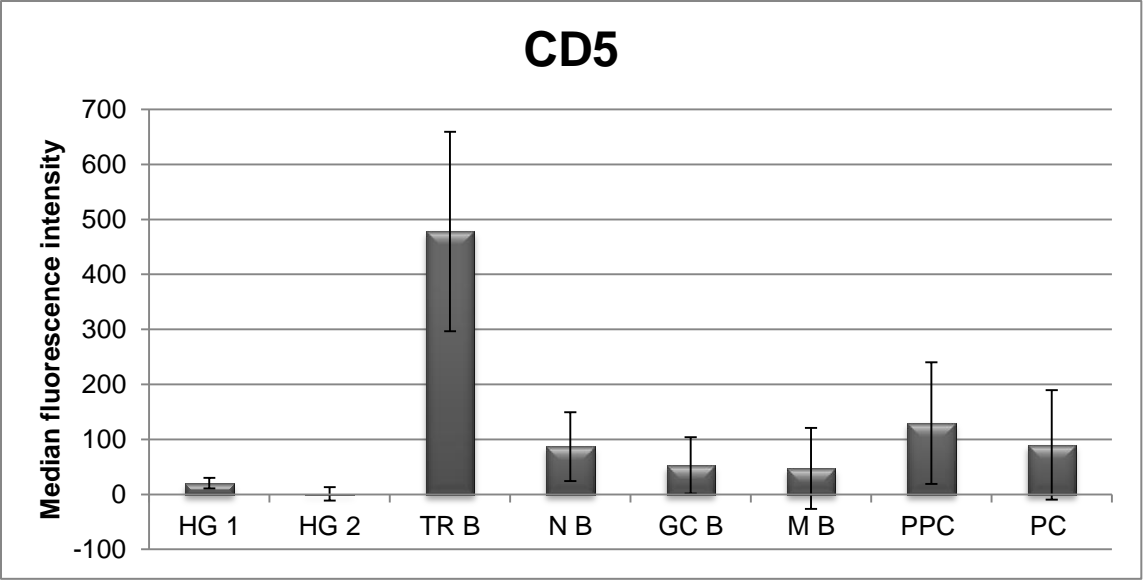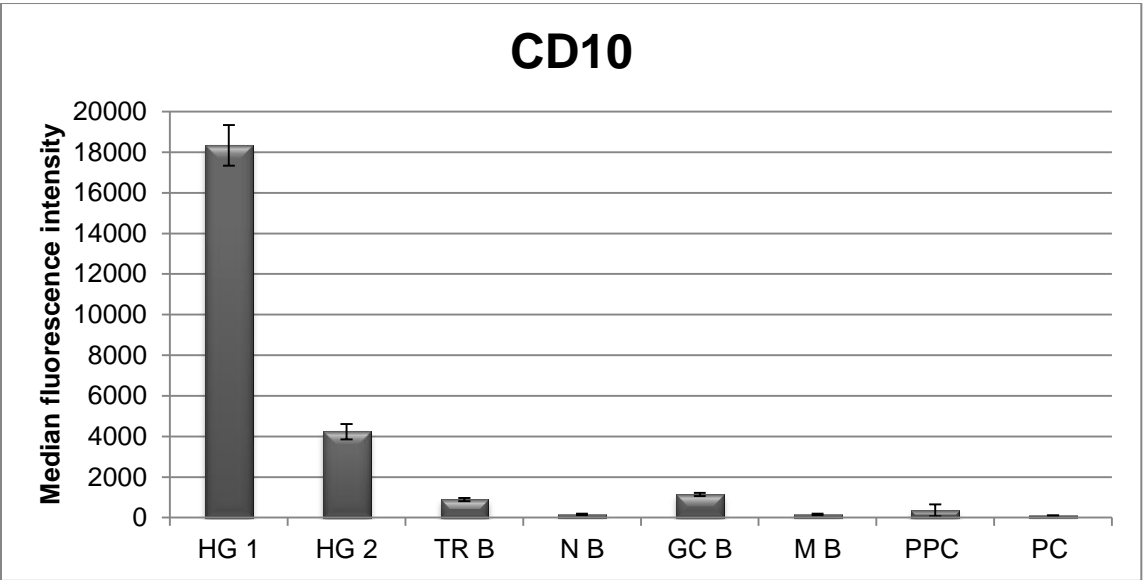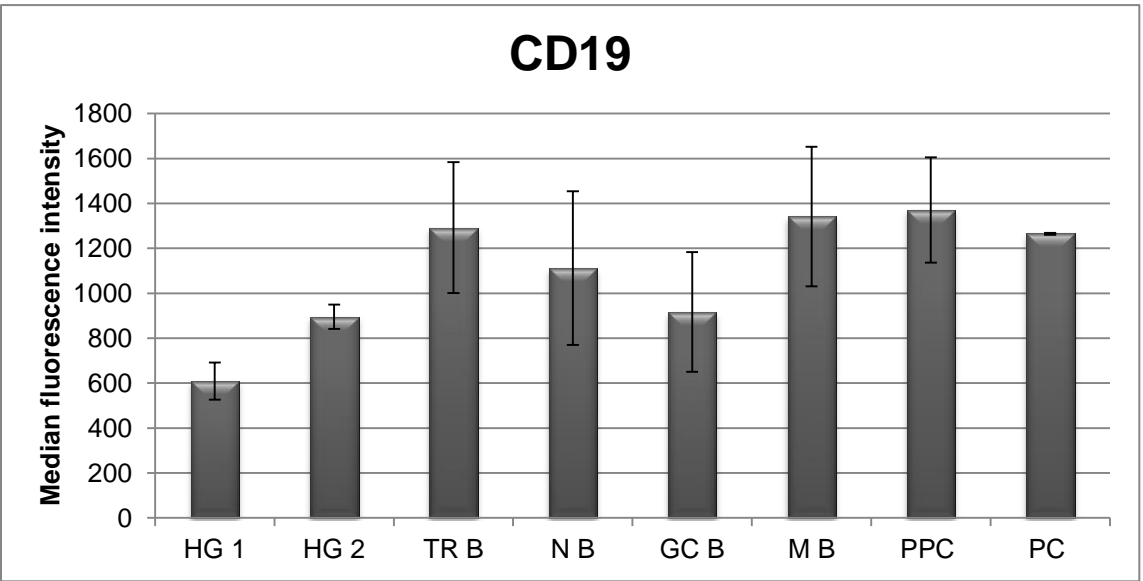

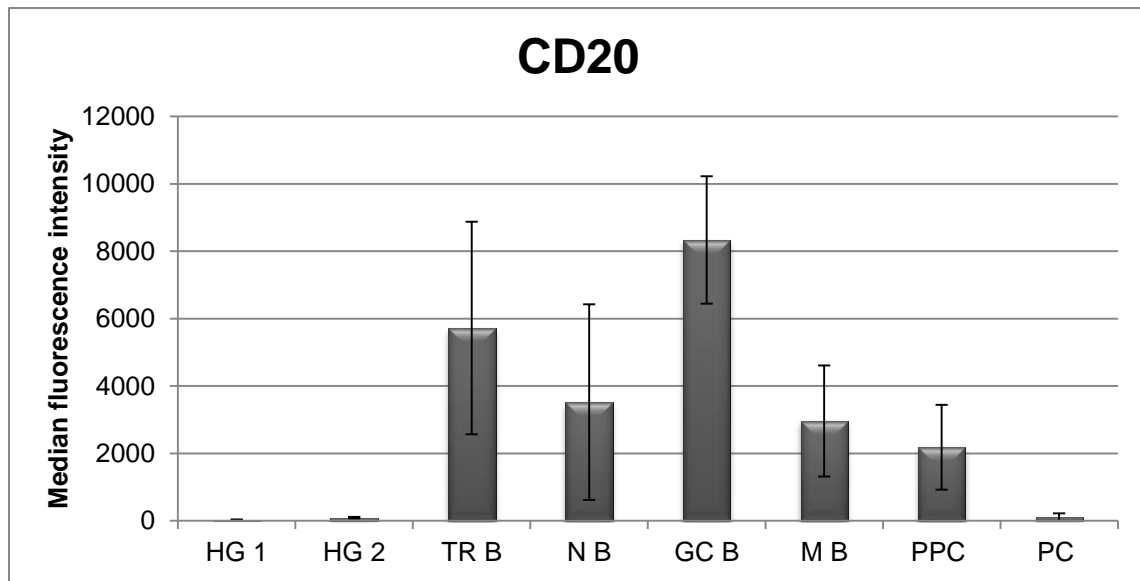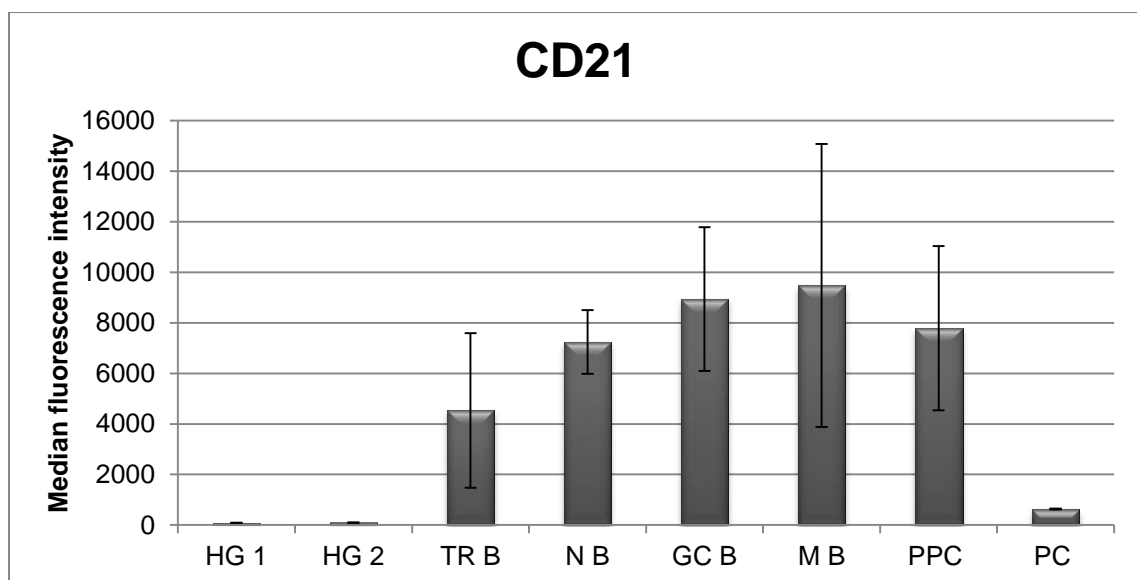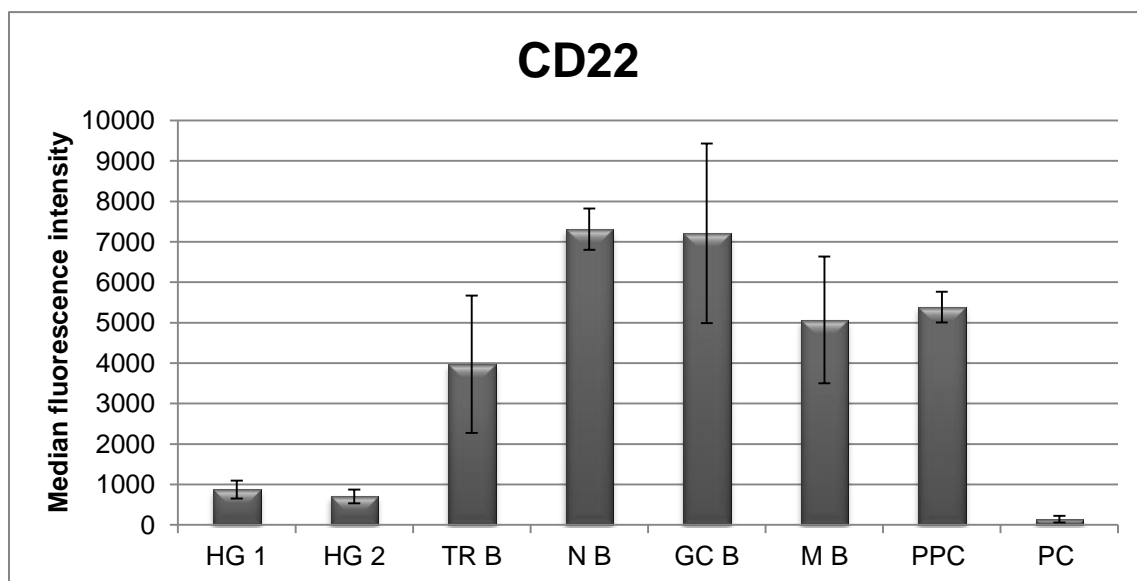

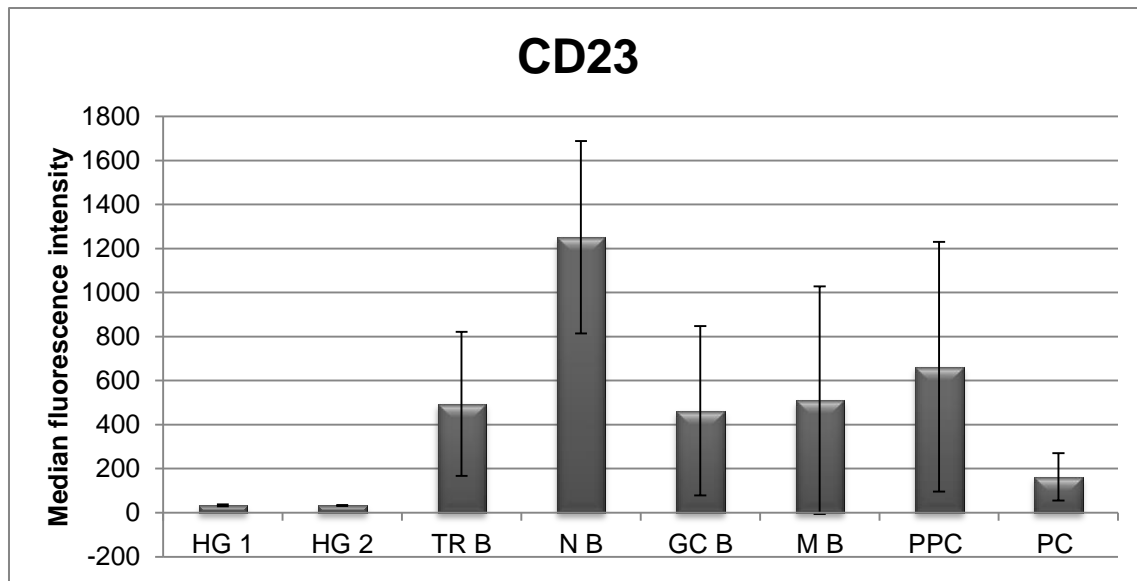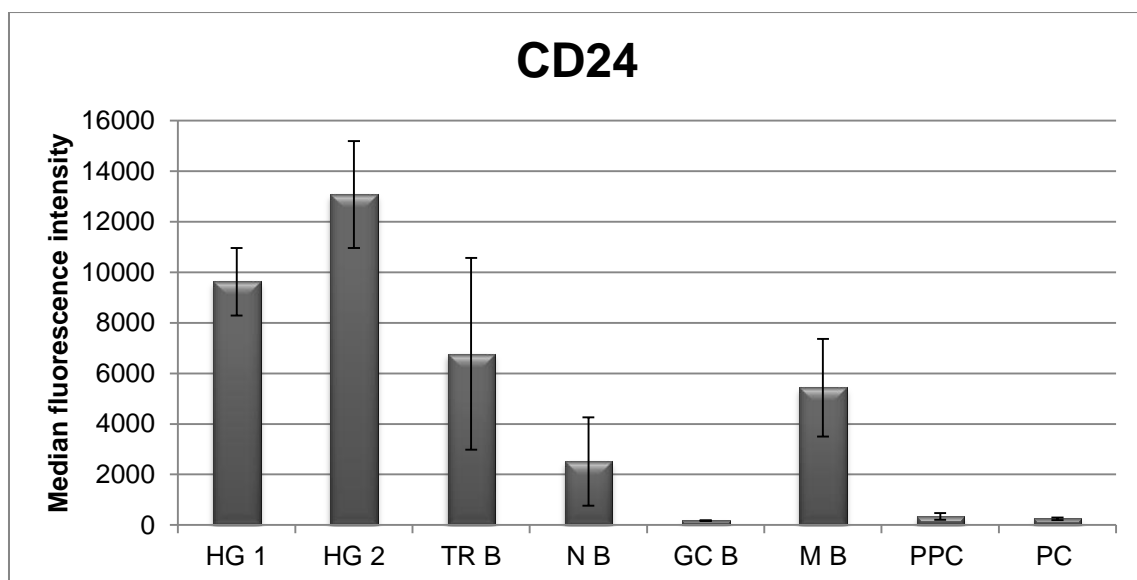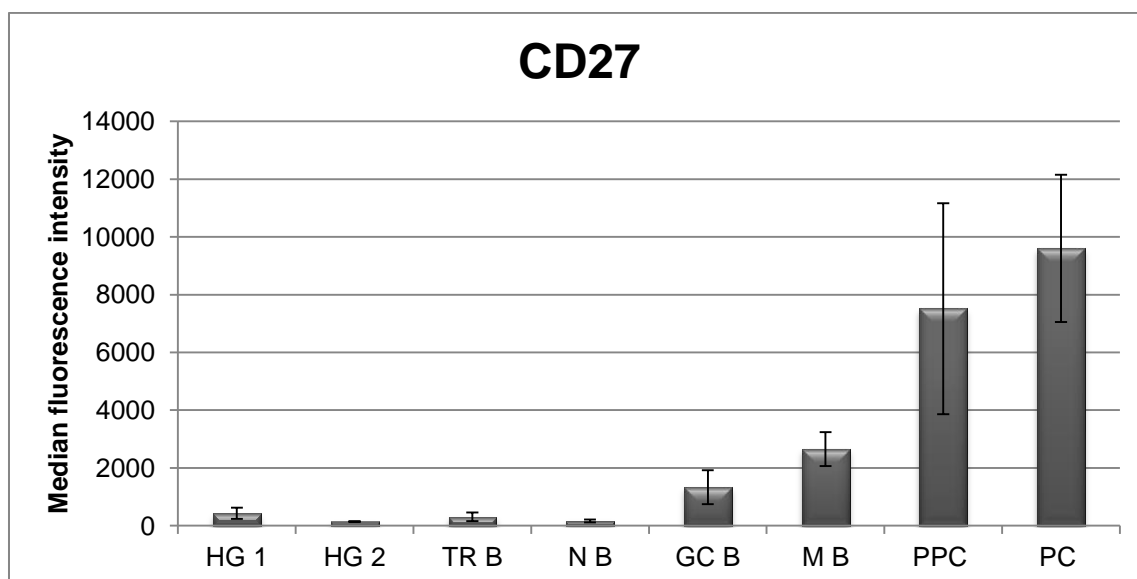

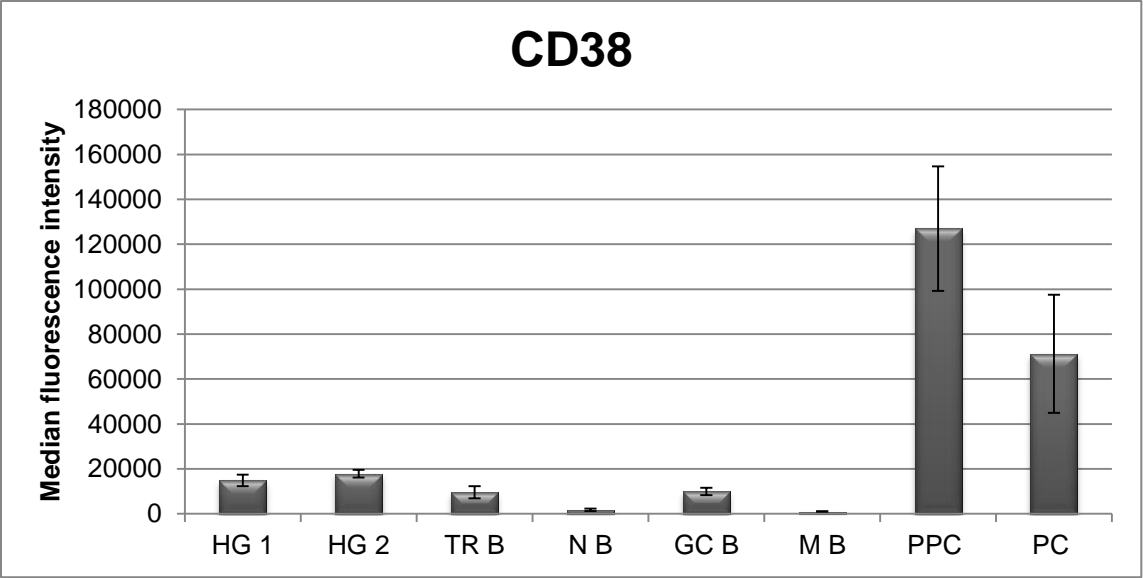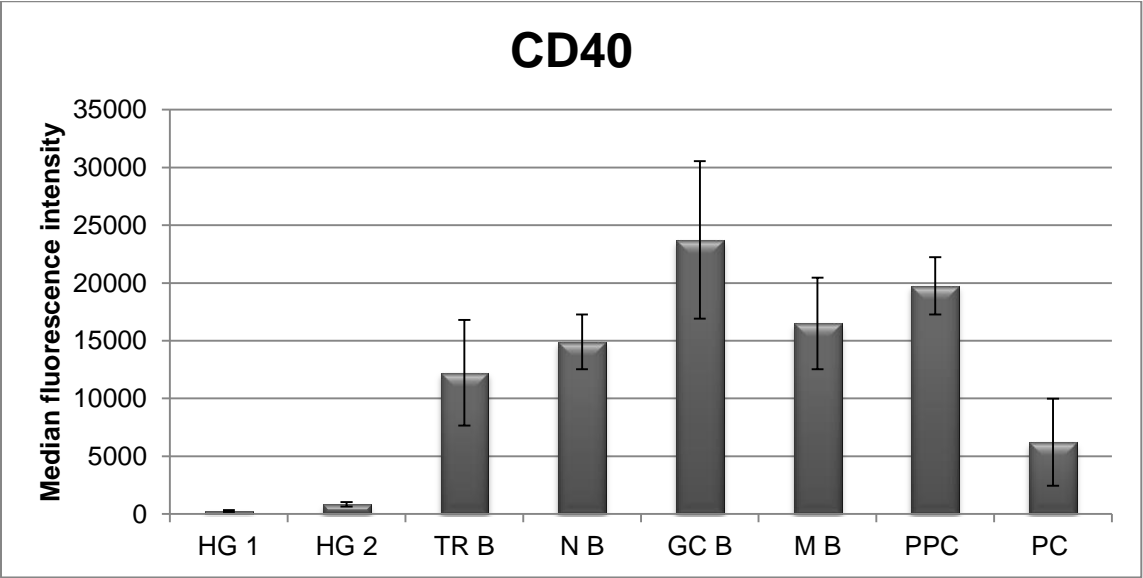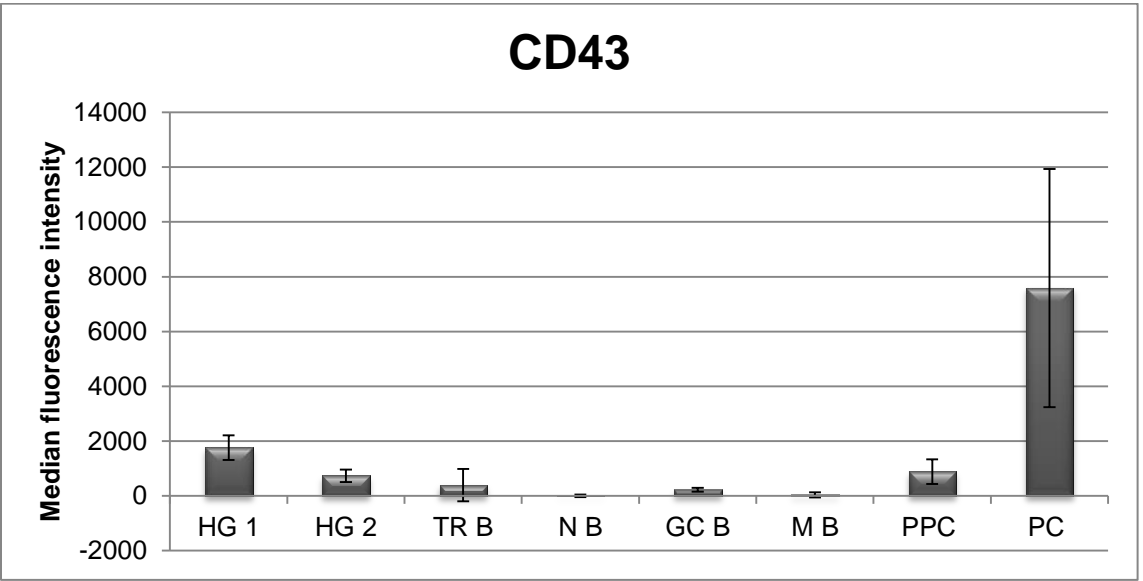

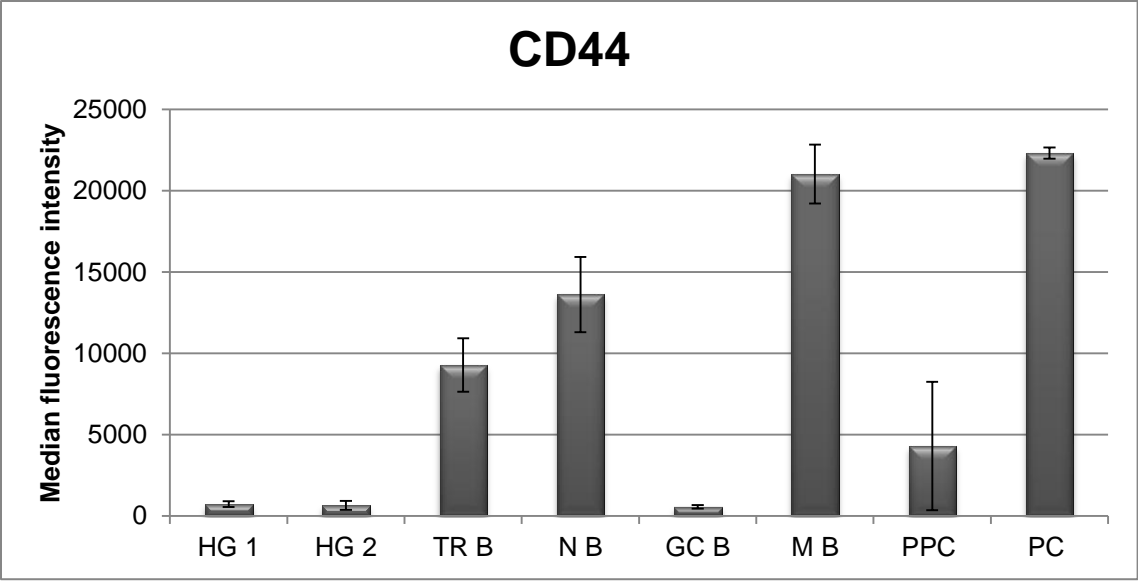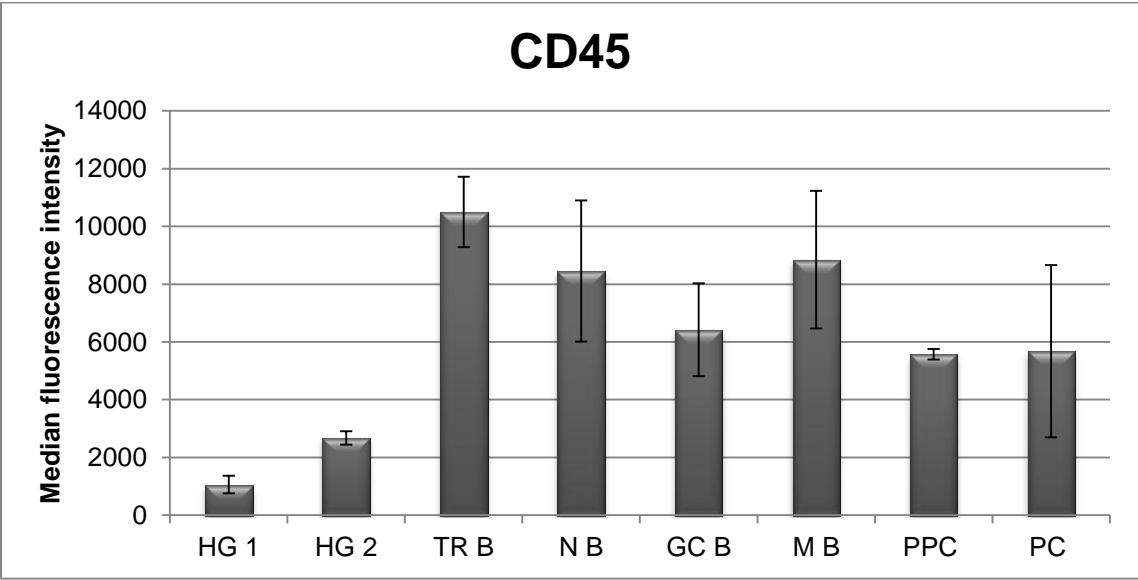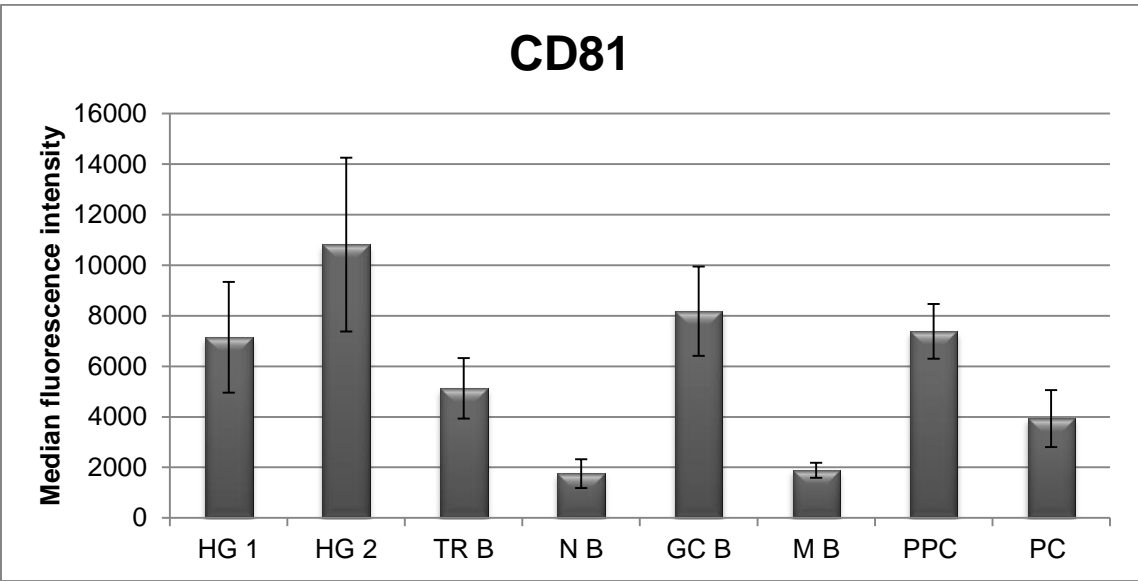

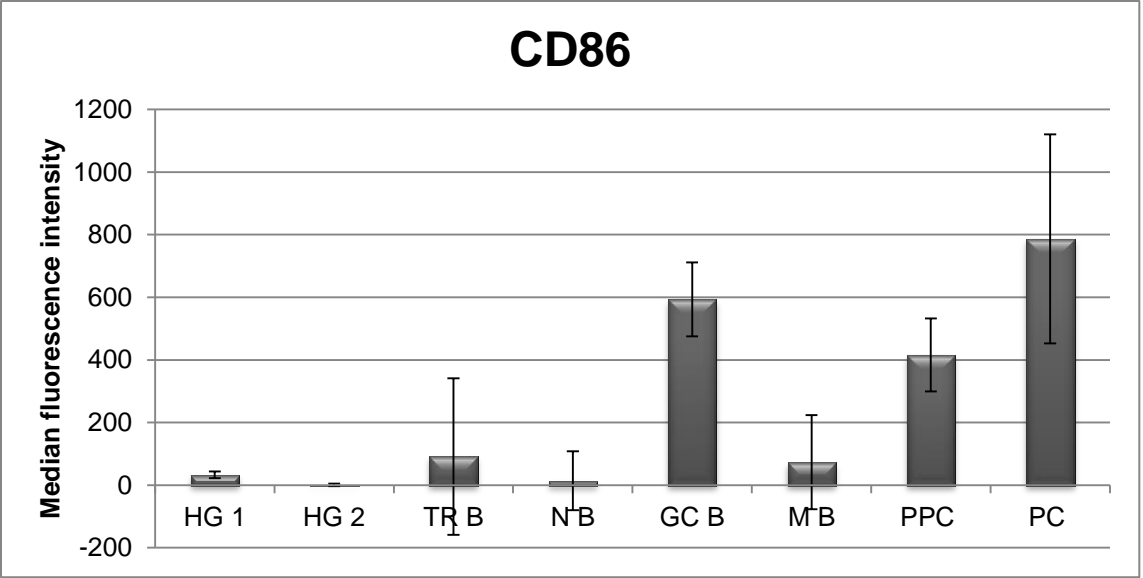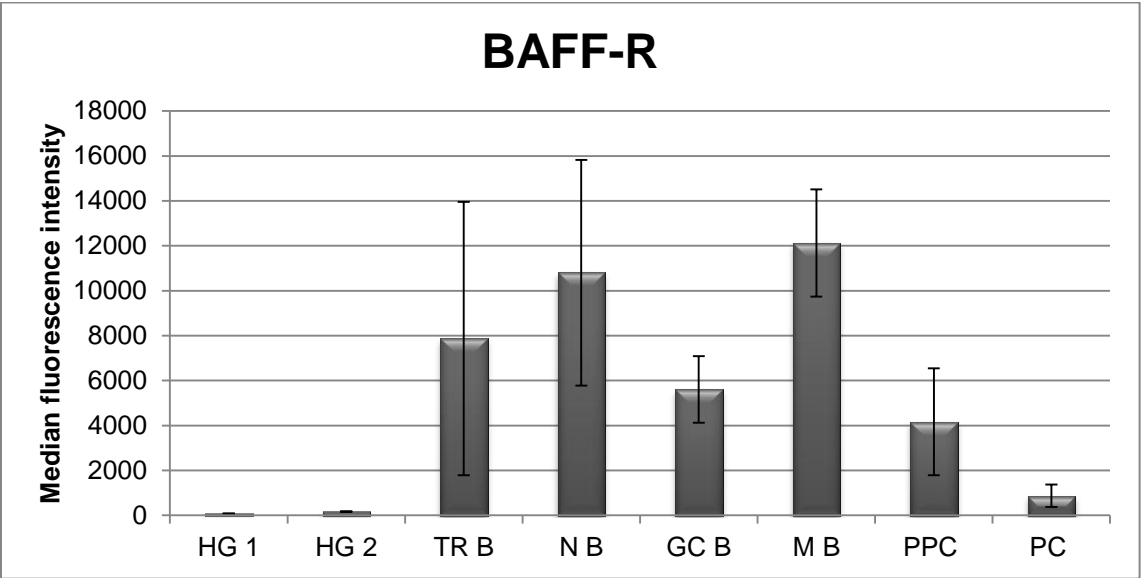

Supplement: S3 Fig — (PDF) [file pone.0162209.s003.pdf]
