# Supplementary material for: Novel Strategy for Phenotypic Characterization of Human B Lymphocytes from Precursors to Effector Cells by Flow Cytometry
Source: PLoS One. 2016 Sep 22;11(9):e0162209. doi: 10.1371/journal.pone.0162209 (PMC5033467; doi:10.1371/journal.pone.0162209)
Supplement: S1 Table — (PDF) [file pone.0162209.s004.pdf]

S1 Table. Characteristics of antibodies.

CD	Fluorochrome	Clone	Isotype	Supplier	Catalogue number
**CD5**	V450	L17F12	Ms IgG2a, κ	BD Biosciences	644487
**CD10**	APC	HI 10a	Ms IgG1, κ	BD Biosciences	332777
**CD19**	APC-H7	SJ25C1	Ms IgG1, κ	BD Biosciences	641395
**CD20**	FITC	B9E9	Ms IgG2a	Beckman Coulter	A07772
**CD21**	PE	B-ly4	Ms IgG1, κ	BD Biosciences	555422
**CD22**	PE	S-HCL-1	Ms IgG2b, κ	BD Biosciences	347577
**CD23**	PE	EBVCS-5	Ms IgG1, κ	BD Biosciences	332782
**CD24**	PE	ALB9	Ms IgG1	Beckman Coulter	IM1428U
**CD27**	PeCy7	1A4CD27	Ms IgG1	Beckman Coulter	A54823
**CD38**	PerCP-Cy5.5	HIT2	Ms IgG1, κ	BD Biosciences	551400
**CD40**	PE	MAB89	Ms IgG1	Beckman Coulter	IM1936U
**CD43**	FITC	1G10	Ms IgG1, κ	BD Biosciences	555475
**CD44**	FITC	J.173	Ms IgG1	Beckman Coulter	IM1219U
**CD45**	V500	HI30	Ms IgG1, κ	BD Biosciences	560777
**CD81**	FITC	JS-81	Ms IgG1, κ	BD Biosciences	551108
**CD86**	FITC	2331(FUN-1)	Ms IgG1, κ	BD Biosciences	555657
**CD268 (BAFF-R)**	PE	11C1	Ms IgG1 κ	BD Biosciences	558097
**IgD**	PE	IA6-2	Ms IgG2a, κ	BD Biosciences	555779
**IgM**	FITC	/	Polyclonal rabbit anti-human	Dako	F005801
